# Thermogravimetric analysis of soot combustion in the presence of ash and soluble organic fraction

**DOI:** 10.1039/d0ra06384c

**Published:** 2020-09-10

**Authors:** Qian Zhang, Jia Fang, Zhongwei Meng, Chen Chen, Zihan Qin

**Affiliations:** Key Laboratory of Fluid and Power Machinery, Ministry of Education, School of Energy and Power Engineering, Xihua University Chengdu 610039 P. R. China jiafang@mail.xhu.edu.cn mengzw@mail.xhu.edu.cn; Vehicle Measurement, Control and Safety Key Laboratory of Sichuan Province, School of Automobile and Transportation, Xihua University Chengdu 610039 P. R. China

## Abstract

Soot (Printex U, PU) combustion in the presence of ash and soluble organic fraction (SOF) was studied by thermogravimetric analysis (TGA). The comprehensive combustion index, combustion stability index and peak temperature were collected to evaluate the combustion performance of soot/ash/SOF mixtures. Compared with SiO_2_, Fe_2_O_3_ and CaSO_4_ nanoparticles, ZnO nanoparticles efficiently accelerate soot combustion with excellent oxygen carrying abilities. When the weight ratio of the PU/ZnO mixture is 1 : 1, this acceleration effect is maximized in the soot combustion process. The comprehensive combustion and combustion stability indices increase from 0.667 × 10^−7^%^2^ min^−2^ °C^−3^ and 23.53 × 10^5^ to 1.296 × 10^−7^%^2^ min^−2^ °C^−3^ and 39.53 × 10^5^, compared to pure PU, respectively. Compared with the PU/ZnO mixture, the soot combustion had inferior results after adding two oils as the simulative SOF. The 15W lubricant had the minimum negative impact compared to 0# diesel fuel. The comprehensive combustion and combustion stability indices reach the maximum values of 1.074 × 10^−7^%^2^ min^−2^ °C^−3^ and 33.29 × 10^5^ at the 1 : 1 : 0.1 weight ratio of PU/ZnO/15W, which grew by 62% and 42% compared to pure PU, respectively. This work contributes to an understanding of the combined effect of ash and SOF on soot combustion.

## Abstract 2

Soot (Printex U, PU) combustion in the presence of ash and soluble organic fraction (SOF) was studied by thermogravimetric analysis (TGA).

## Graphical abstract

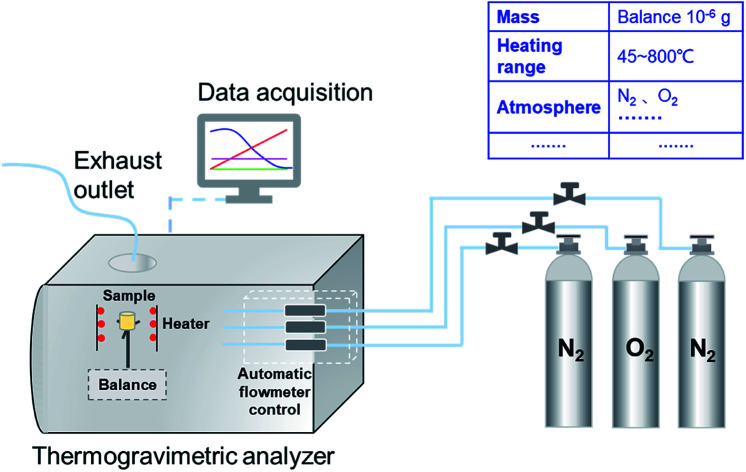

## 1. Introduction

In recent years, energy consumption and environmental protection have been hot topics,^1^ which places particulate matter (PM) in the spotlight. There are many scholars from different academic fields devoted to conducting overarching studies on this topic.^2–4^ The survey results indicate that PM not only results in air pollution,^5,6^ but contributes to health problems^7^ such as respiratory and immune system problems,^8^ obesity,^9^ chronic kidney disease^10^ and cardiovascular dysfunctions.^11^ Specifically, fine PM2.5 (particles with an aerodynamic diameter ≤ 2.5 μm) is linked to cell death^12^ and lung cancer because once the harmful particles enter cellular tissue, they cannot be discharged.^13^ As one of the main sources of air contamination, the prevalent diesel engine is a contributor of PM^14^ in spite of its high thermal efficiency, reliability and fuel economy.^15^ In order to balance the increasingly restrictive regulations on vehicle exhaust, techniques to purify diesel emission have become diverse,^16^ such as exhaust gas recirculation (EGR),^17^ diesel particulate filtering (DPF),^18^ selective catalyst reduction (SCR)^19^ and diesel oxidation catalysis (DOC).^20^ Among them, DPF is considered the most effective technology towards PM reduction with a capturing efficiency of more than 95%.^21^ However, the excessive soot loadings result in a high pressure drop, fuel penalty^21^ and DPF degradation,^22^ so periodic regeneration is inevitable.^23^ Therefore, a great deal of effort has been devoted to investigating the regeneration process for the extension of DPF life time and emission reduction.^24,25^

Initially, it was reported that PM mainly contains dry soot, SOF, sulfates, ash, and moisture.^26^ Similarly, Mohankumar *et al.*^27^ proposed that PM could be divided into a soluble and insoluble organic fraction, and soot accounts for the major proportion. Liati *et al.*^28,29^ analyzed the ash chemical composition, which consisted mainly of Ca, Mg, P, Zn, S, O and minor amounts of Fe, Al and Si. Moreover, Jiang *et al.*^30^ reported that ash was an inorganic and non-combustible fraction, and the over-loading of ash would cause the filtration efficiency, flow resistance and service life of DPF to deteriorate. Furthermore, the effects of the components of PM have also been investigated. Fang *et al.*^31^ used a thermogravimetric (TG) analyzer to study the interaction effect of catalyst and ash on diesel soot oxidation, and the results showed that a 1 : 5 : 5 weight ratio of a soot/CeO_2_/ZnO mixture had the best combustion performance under an O_2_/N_2_ atmosphere. Collura *et al.*^32^ stated that the release of thermally labile groups of soot and the confined decomposition of the non-volatile part of SOF on the soot surface led to an increase in the specific surface area of PM. Xu *et al.*^33^ found that the particles had difficulty oxidizing due to the increasing microcrystalline length of carbon smoke particles and that the carbon layer structure improved after the combustion of the lubricating oil.

All of the above studies have largely been focused on soot oxidation and the influence of ash or SOF alone on soot combustion. Yet, the interaction effect of ash with SOF on the soot combustion process is still unclear, which is equally inevitable towards the regeneration process. The combustion characteristics of PU, PU/ash and PU/ash/SOF mixtures with different ash species, SOF types and their corresponding proportions are presented in this paper. In this context, the mechanism of soot oxidization in the presence of ash and SOF is investigated through a series of TG experiments. The objective of this work is to lay a theoretical foundation to further understand the combustion characteristics of diesel soot in the presence of ash and SOF.

## 2. Experimental study

### 2.1 Materials and samples preparation

As the typical commercial synthetic soot, PU employed in this study is sourced from Degussa Gmbh, which is able to maintain high experimental repeatability^34,35^ and the detailed parameters are listed in Table 1. To simulate the major ash contents of exhaust soot discharged from the diesel engine, four kinds of ash were adopted, including ZnO, SiO_2_, Fe_2_O_3_, and CaSO_4_.^17,36^ ZnO and CaSO_4_ ashes were provided by Shanghai Chaowei Nanotechnology and Alfa Aesar, respectively, while the other ashes were supplied by Shanghai Aladdin Biochemical. The ash particle size mostly varies from 30 to 60 nm, so 30 nm and 50 nm ashes were introduced in the consequent experiments.^29^ The specifics of the ashes are shown in Table 2. The 0# diesel fuel and 15W lubricant commonly used in the diesel engine were chosen as alternatives to SOF because they are proven to be the main sources of SOF.^32,37,38^

**Table 1:** Detailed parameters of PU

Soot	Average diameter (nm)	BET (m^2^ g^−1^)	Oil absorption (g/100 g)	Ash content (%)
PU	25	92	460	0.02

**Table 2:** Physical properties of ashes

Serial number	Contents	Average diameter (nm)	Metals basis
1#	ZnO	50 ± 10	99.8%
2#	SiO_2_	30	99.9%
3#	Fe_2_O_3_	50	99.5%
4#	CaSO_4_	50	99.9%

In order to obtain the PU/ash and PU/ash/SOF mixtures as the desired samples, an electronic balance with a high accuracy of 10^−4^ g was applied to measure the weight of the raw powders. Then, all the samples were dried at 110 °C for 24 h in a vacuum oven to remove the moisture. Afterwards, each sample was fully shaken in a vortex mixer three times and each duration was about 10 min to obtain a good uniformity.

### 2.2 Apparatus

**Fig. 1:**
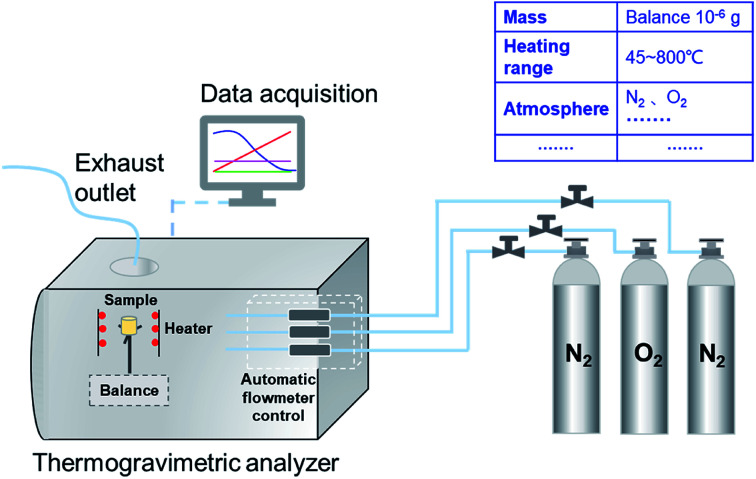
The schematic diagram of TG209F3.

The combustion characteristics of the prepared samples were analyzed through the TG analyzer (TG209F3, NETZSCH, Germany), as shown in Fig. 1. The temperature precision of the analyzer was ±0.1 °C and its microbalance sensitivity was less than 0.1 μg, which ensures the experimental data accuracy. The weight of PU was fixed at 3 mg in each test. Then, the sample was placed in the alumina crucible (diameter × height: 6.8 × 7.4 mm) and heated from 45 to 800 °C at the ramp rate of 10 °C min^−1^. There was a 10 min holding time at the beginning and the end of each test to ascertain the stability of the instrument. The oxygen and nitrogen were fed in with a 100 mL min^−1^ flow rate in the ratio of 1 : 9 as the carrier gases. Then, a benchmark experiment with an empty crucible was performed in order to eliminate the TG curve drift resulting from the buoyancy and other factors.^39,40^ All the tests were carried out twice, and the repeatability and reproducibility of the results were high.

### 2.3 Data analysis

**Fig. 2:**
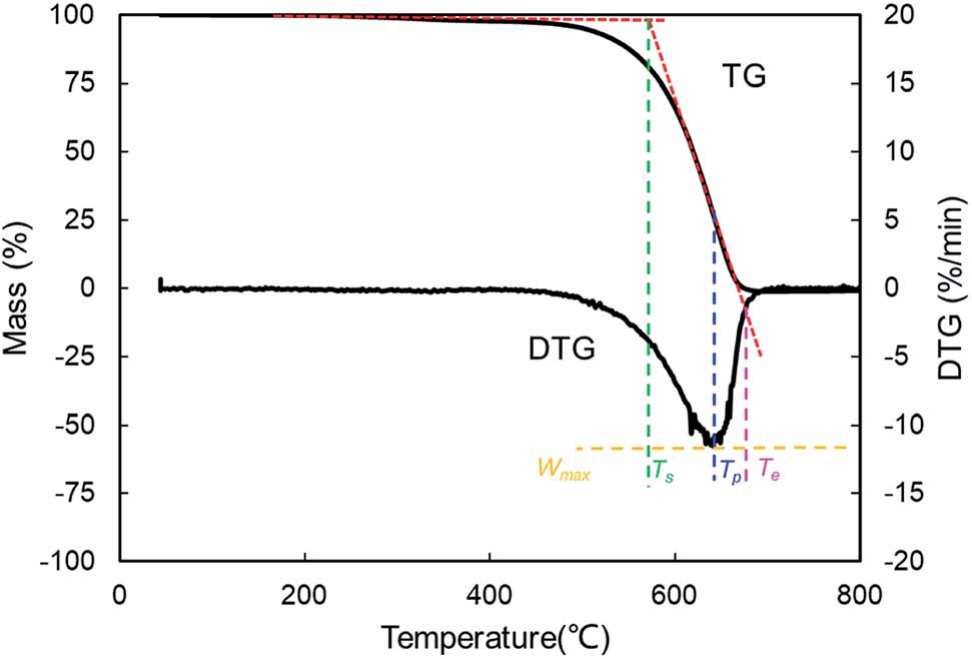
The definition of combustion parameters with the TG–DTG tangent method.

Based on the recorded TG and the derivative thermogravimetric (DTG) profiles, the starting temperature (*T*_s_), ending temperature (*T*_e_), peak temperature (*T*_p_), maximum mass loss rate (*W*_max_) and mean mass loss rate (*W*_mean_) were extracted to evaluate the combustion performance. Particularly, *T*_s_ refers to the point where the sample begins to burn and *T*_e_ is defined as the temperature when the elementary combustion process is realized. *T*_p_ represents the corresponding temperature to the peak of the DTG outline, which represents *W*_max_. The average value of mass loss rate from the beginning of the reaction to end is *W*_mean_.^41–43^ These combustion parameters can be described appropriately by the TG–DTG tangent method as described in Fig. 2.

To fully assess the combustion performance, some vital combustion indices were introduced, such as the comprehensive combustion index (*S*) and combustion stability index (*R*_w_).

The ignition, combustion and burnout properties can be estimated by the *S* index, and the calculation is as follows:1
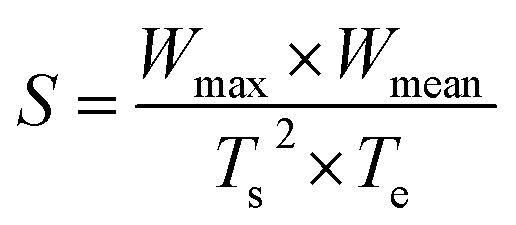
where *W*_max_, *W*_mean_, *T*_s_ and *T*_e_ represent the maximum mass loss rate, the mean mass loss rate, the starting temperature and the ending temperature, respectively. Higher values of the *S* index indicate that the combustion performance is better.^43^

The *R*_w_ index reflects the stability in the process of combustion,^44^ which is defined as:2
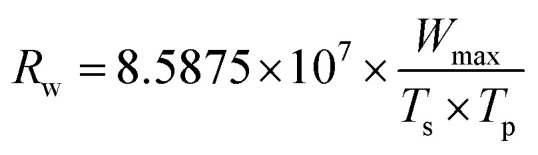
where *W*_max_, *T*_s_ and *T*_p_ represent the maximum mass loss rate, the starting temperature and the peak temperature, respectively.

To facilitate comparison of the combustion performance among the samples, the characteristic parameters of all 32 cases are summarized in Table 3.

**Table 3:** The Summary of characteristic parameters of all samples in this study

Case #	Soot/ash/SOF	Blending ratio	*T*_s_ (°C)	*T*_e_ (°C)	*T*_p_ (°C)	*W*_mean_ (% min^−1^)	*W*_max_ (% min^−1^)	*S* × 10^7^ (%^2^ min^−2^ °C^−3^)	*R*_w_ (10^5^)
1–2	PU : — : —	—	577 ± 2	697 ± 1	648 ± 3	1.51 ± 0.01	10.25 ± 0.32	0.667 ± 0.023	23.53 ± 0.77
3–4	PU : SiO_2_ : —	1 : 1 : —	573 ± 2	682 ± 2	642 ± 3	1.55 ± 0.01	11.26 ± 0.01	0.779 ± 0.004	26.27 ± 0.21
5–6	PU : Fe_2_O_3_ : —	1 : 1 : —	565 ± 0	687 ± 1	640 ± 1	1.54 ± 0.00	10.02 ± 0.11	0.702 ± 0.005	23.78 ± 0.25
7–8	PU : ZnO : —	1 : 1 : —	552 ± 3	636 ± 0	595 ± 4	1.67 ± 0.01	15.11 ± 0.25	1.296 ± 0.01	39.53 ± 0.21
9–10	PU : CaSO_4_ : —	1 : 1 : —	587 ± 1	692 ± 1	654 ± 1	1.54 ± 0.01	11.30 ± 0.21	0.730 ± 0.015	25.28 ± 0.46
11–12	PU : ZnO : —	1 : 0.5 : —	552 ± 2	641 ± 0	600 ± 2	1.64 ± 0.01	14.09 ± 0.13	1.182 ± 0.003	36.53 ± 0.16
13–14	PU : ZnO : —	1 : 0.7 : —	553 ± 5	648 ± 4	599 ± 3	1.61 ± 0.01	13.68 ± 1.38	1.113 ± 0.101	35.49 ± 3.14
15–16	PU : ZnO : —	1 : 5 : —	538 ± 5	631 ± 4	596 ± 4	1.64 ± 0.02	12.73 ± 0.30	1.145 ± 0.013	34.07 ± 0.30
17–18	PU : ZnO : —	1 : 7 : —	527 ± 2	626 ± 2	590 ± 1	1.62 ± 0.01	11.56 ± 0.23	1.079 ± 0.015	31.94 ± 0.47
19–20	PU : ZnO : —	1 : 15 : —	511 ± 3	623 ± 1	586 ± 1	1.60 ± 0.01	10.13 ± 0.35	0.998 ± 0.028	29.04 ± 0.87
21–22	PU : ZnO : 0#	1 : 1 : 0.2	552 ± 0	651 ± 1	606 ± 3	1.61 ± 0.01	12.45 ± 0.23	1.009 ± 0.014	31.93 ± 0.73
23–24	PU : ZnO : 15W	1 : 1 : 0.2	529 ± 3	635 ± 1	600 ± 1	1.64 ± 0.01	11.10 ± 0.17	1.024 ± 0.005	30.05 ± 0.28
25–26	PU : ZnO : 15W	1 : 1 : 0.05	542 ± 2	644 ± 2	597 ± 0	1.60 ± 0.02	12.01 ± 0.58	1.018 ± 0.039	31.90 ± 1.47
27–28	PU : ZnO : 15W	1 : 1 : 0.1	547 ± 4	639 ± 4	601 ± 1	1.61 ± 0.02	12.74 ± 0.20	1.074 ± 0.021	33.29 ± 0.26
29–30	PU : ZnO : 15W	1 : 1 : 0.4	552 ± 1	645 ± 1	603 ± 1	1.62 ± 0.01	10.41 ± 0.05	0.860 ± 0.002	26.87 ± 0.17
31–32	PU : ZnO : 15W	1 : 1 : 1	529 ± 2	630 ± 3	602 ± 1	1.66 ± 0.01	6.86 ± 0.04	0.647 ± 0.009	18.51 ± 0.01

## 3. Results and discussion

### 3.1 Influence of ash species on soot combustion

**Fig. 3:**
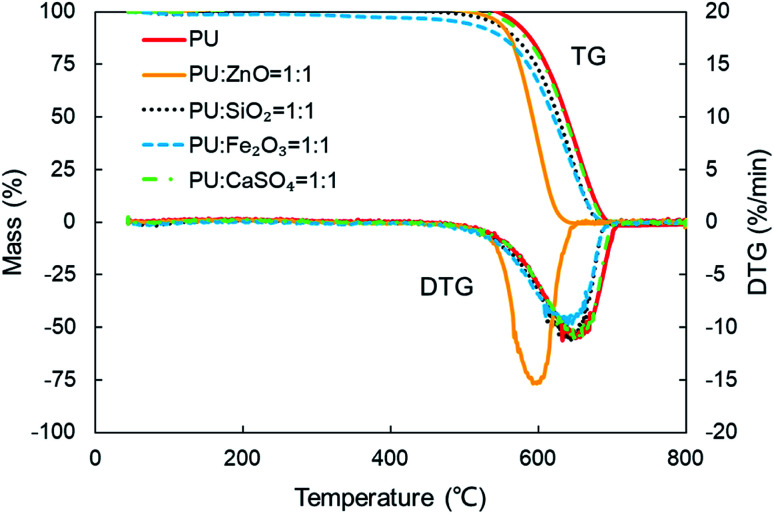
TG and DTG profiles of combustion for pure PU and different soot/ash mixtures at blending ratio of 1 : 1.

**Fig. 4:**
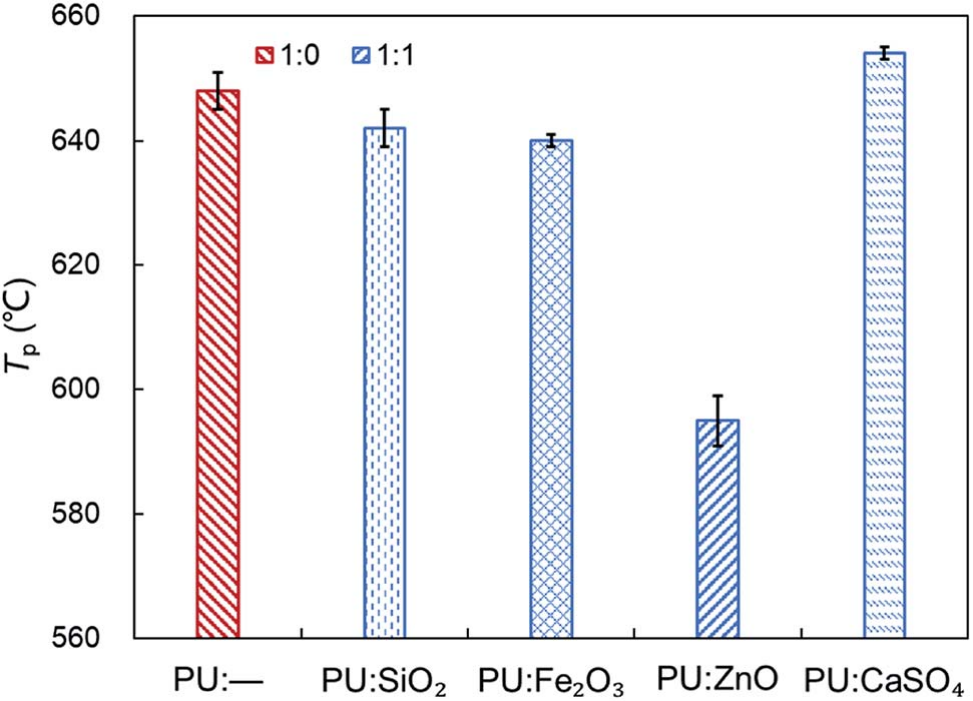
Comparison of the *T*_p_ among pure PU and different soot/ash mixtures at blending ratio of 1 : 1.

**Fig. 5:**
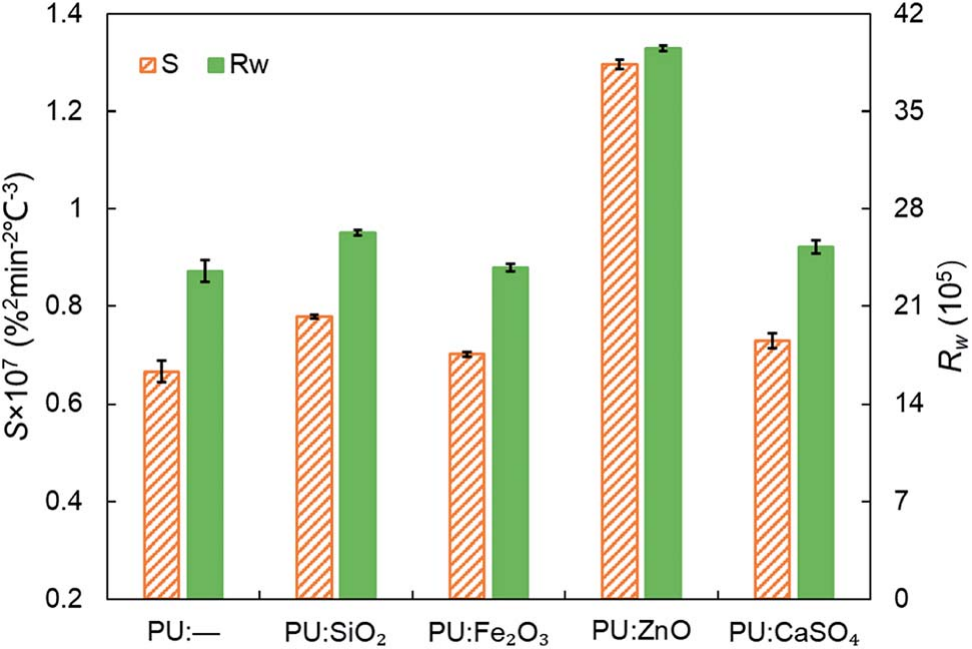
Comparison of the *S* and *R*_w_ indices among pure PU and different soot/ash mixtures at blending ratio of 1 : 1.

In order to study the influence of ash species on soot oxidation, several oxides, such as ZnO, SiO_2_, Fe_2_O_3_, and CaSO_4_, were selected to conduct the experiments at the heating rate of 10 °C min^−1^ in a 10% O_2_/90% N_2_ atmosphere. As illustrated in Fig. 3, the PU oxidation processes caused by different ashes are distinctive. After adding the ZnO nanoparticles, an advance oxidation of PU is observed appreciably from the TG and DTG profiles. There is little change in the PU oxidation process with the addition of SiO_2_, Fe_2_O_3_ and CaSO_4_ nanoparticles. In particular, the TG and DTG profiles of the PU/CaSO_4_ mixture almost match that of pure PU, while the starting and peak temperatures of PU/CaSO_4_ present a negative effect compared to pure PU. Additionally, the *S* and *R*_w_ indices of PU/Fe_2_O_3_ are close to that of pure PU according to Table 3. As shown in Fig. 4, the peak temperature *T*_p_ of pure PU is approximately 650 °C when the oxygen concentration is 10%. Whereas, Fang *et al.*^31^ found that the *T*_p_ of pure PU is about 620 °C when the oxygen concentration is 20%. It indicates that the high oxygen concentration is beneficial for soot combustion. After blending the equal proportion of ZnO, the *T*_p_ of PU decreases to 595 °C. It implies that ZnO enables the largest mass loss of PU to happen in advance. Likewise, a similar trend also appears in Fig. 5. The value of the comprehensive combustion index *S* of the PU/ZnO mixture reached 1.296 × 10^−7^%^2^ min^−2^ °C^−3^, which is twice that of pure PU. Meanwhile, the combustion stability index *R*_w_ increases to 39.53 × 10^5^, which increases 70% compared with that of pure PU. ZnO as a transition metal oxide is a great oxygen carrier that is able to promote soot combustion effectively. The mechanism of soot oxidation reactions in C–ZnO–O_2_ proposed by Fang *et al.* are explained as follows.^45^

3 ZnO + C → Zn + CO

4 2ZnO + C → 2Zn + CO_2_

5 2Zn + O_2_ → 2ZnO

Another possible reason is that ZnO has a larger surface area and more active sites exist on the outer surface than on SiO_2_, CaSO_4_ and Fe_2_O_3_. Thus, it is beneficial to combine oxygen molecules and make soot more easy to oxidize based on the study of Nascimento *et al.*^46^ On account of the promotional effect on soot combustion, ZnO was used as the ash in subsequent experiments.

### 3.2 Influence of ash blending ratio on soot combustion

**Fig. 6:**
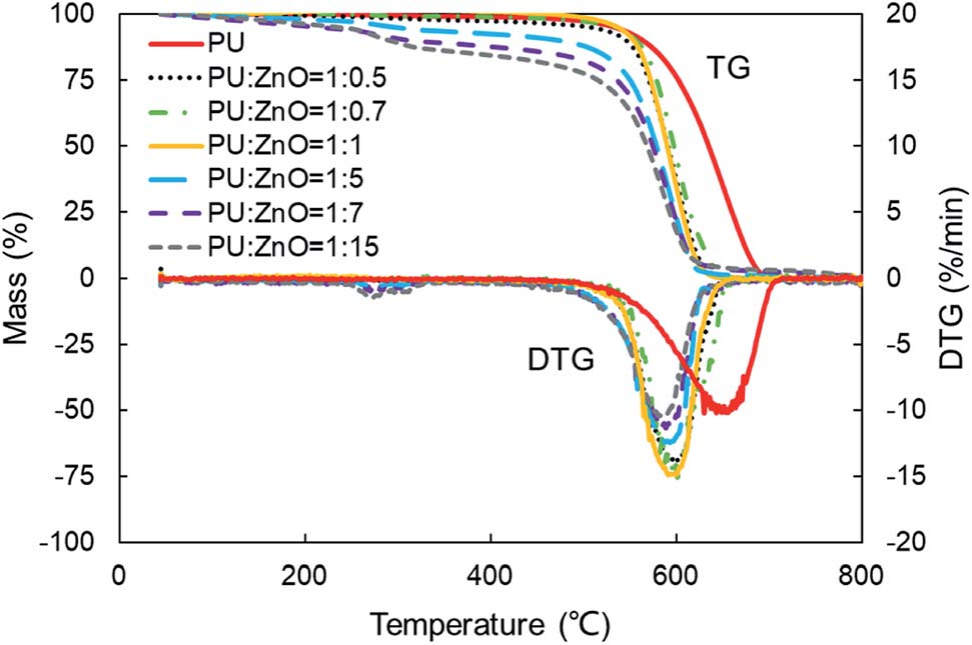
TG and DTG profiles of combustion for soot/ash mixtures at different blending ratios.

**Fig. 7:**
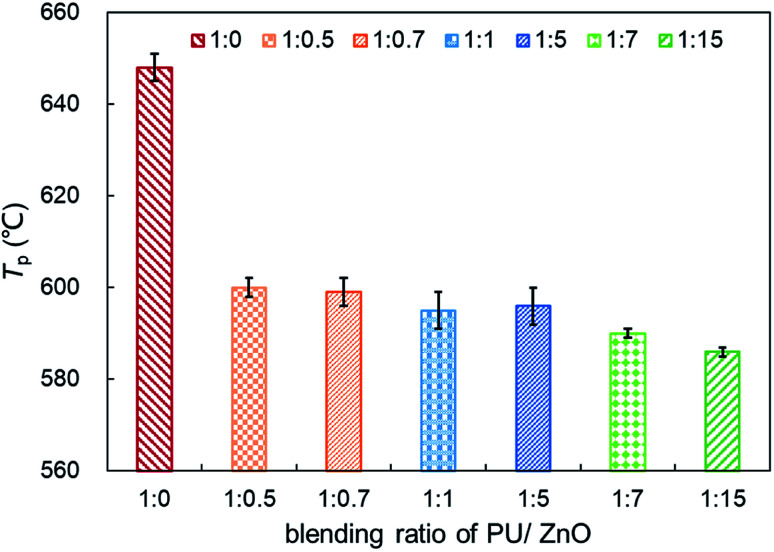
Comparison of the *T*_p_ of soot/ash mixtures at different blending ratios.

**Fig. 8:**
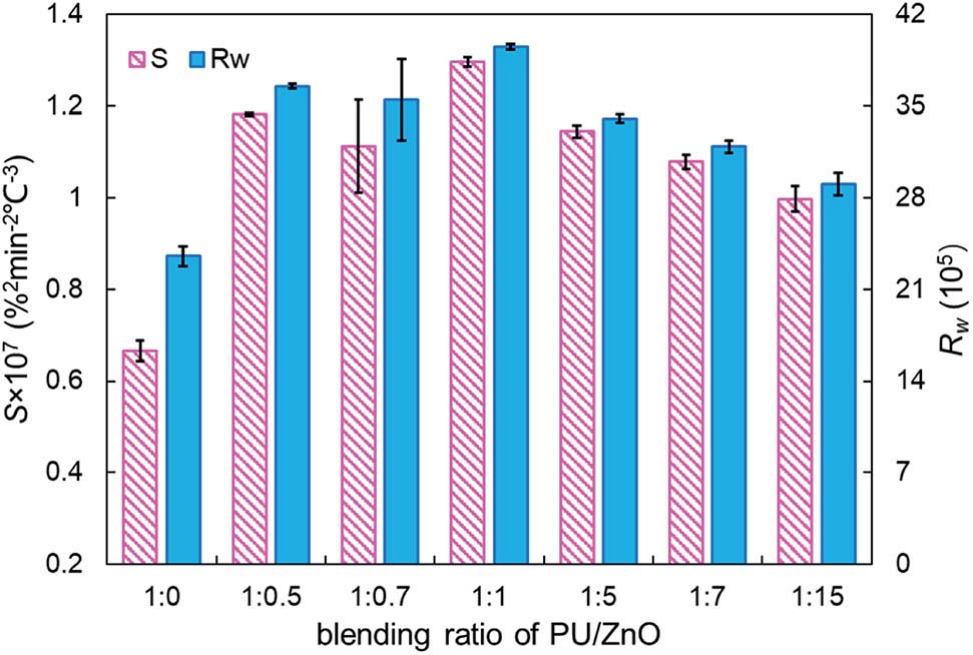
Comparison of the *S* and *R*_w_ indices of soot/ash mixtures at different blending ratios.

Fig. 6 displays the TG and DTG curves of soot/ash mixtures with various blending ratios of ash. Both TG and DTG curves drift to the lower temperature zone as the blending ratio of ZnO increases in the PU/ZnO mixture. However, the value of *W*_max_ increases first and then decreases when the proportion of PU/ZnO varies from 1 : 0.5 to 1 : 15. When the blending ratio of the PU/ZnO mixture is 1 : 1, the maximum value of *W*_max_ reaches 15.11% min^−1^ because the excessive ash particles effectively hide the soot surface and further induce poor heat transfer. From Fig. 7, the increasing proportion of ZnO leads to a moderate decrease in the peak temperature *T*_p_ when the blending ratio of PU/ZnO is below 1 : 15. This may be explained by the fact that more ZnO leads to richer active oxygen sites. As a result, soot particles tend to ignite at a lower temperature, which is conducive to the regeneration process. From another aspect, more ZnO is not always better because the overloaded ZnO can induce unstable soot combustion. Fig. 8 compares the *S* and *R*_w_ indices of PU/ZnO mixtures at blending ratios ranging from 1 : 0 to 1 : 15. The two comprehensive indices present the overall trend that it increases initially, followed by decrease. When the blending ratio of PU/ZnO reaches 1 : 1, both the *S* and *R*_w_ indices reach the maximum, which confirms that the surplus ash nanoparticles do not aid the soot combustion process even if ZnO nanoparticles are good oxygen carriers. Therefore, the combustion performance of PU is promoted considerably at the 1 : 1 weight ratio of PU/ZnO, so this ratio was used in the followed experiments.

### 3.3 Influence of SOF types on soot combustion

**Fig. 9:**
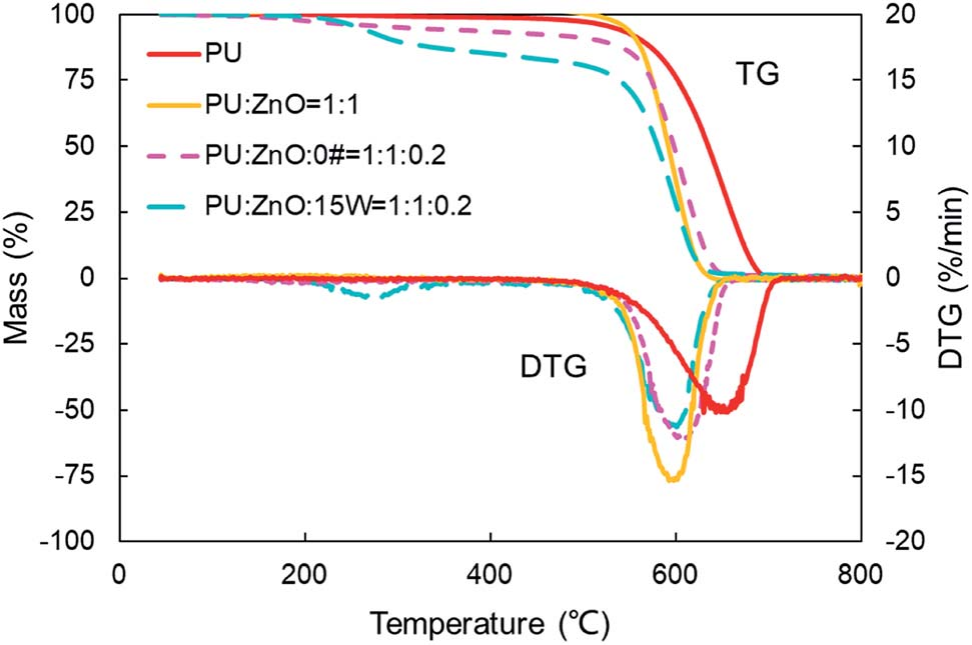
TG and DTG curves of combustion for different soot/ash/SOF mixtures.

**Fig. 10:**
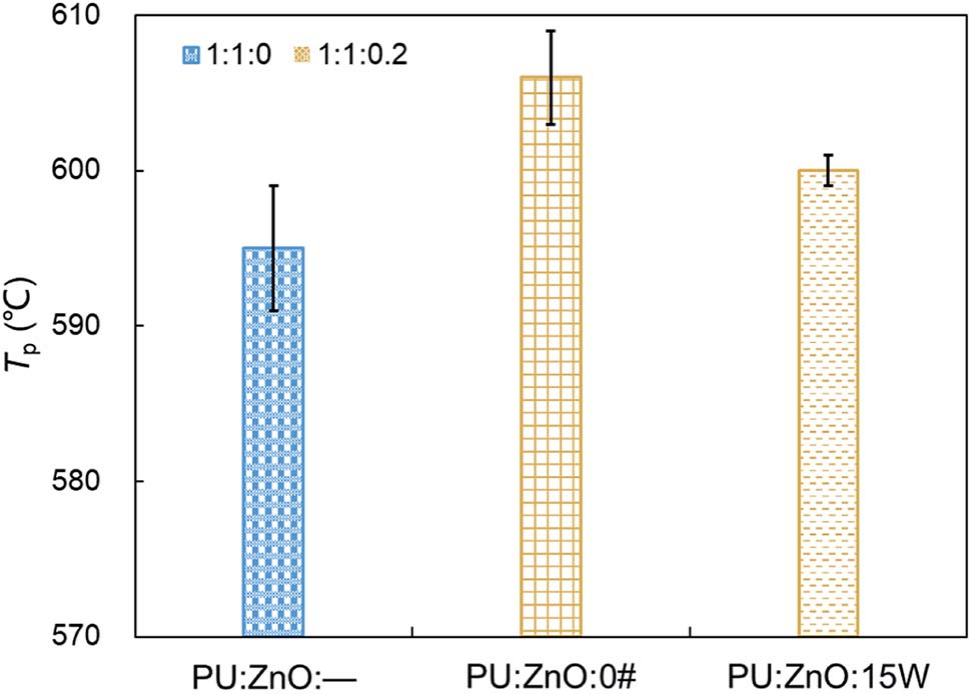
Comparison of the peak temperature *T*_p_ among different soot/ash/SOF mixtures.

**Fig. 11:**
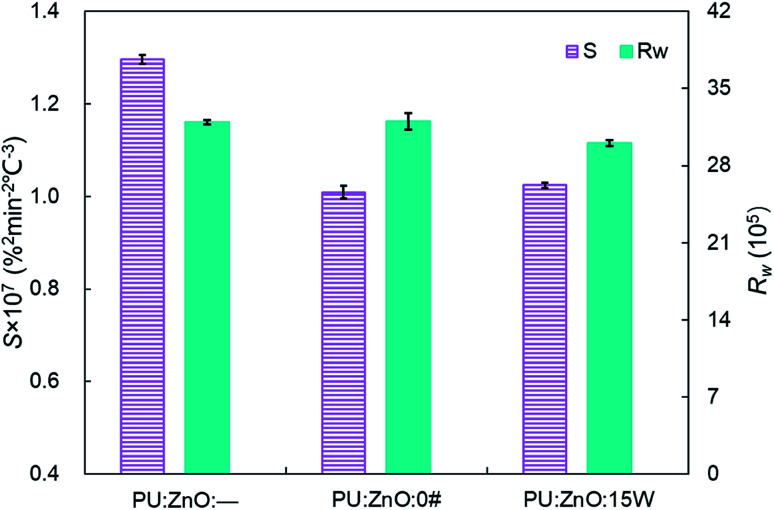
Comparison of the *S* and *R*_w_ indices among different soot/ash/SOF mixtures at blending ratios of 1 : 1 : 0 and 1 : 1 : 0.2.

In this section, the combustion process of soot mixed with ash was further explored through the addition of the SOF. To compare the effect of SOF types on soot combustion, 0# diesel fuel and 15W lubricant as SOF were chosen to blend with the 1 : 1 weight ratio of the PU/ZnO mixture. On average, SOF accounts for 16% of PM emission in a recent study,^47^ so a 1 : 1 : 0.2 weight ratio of soot/ash/SOF was employed. The tendency of the mass loss and its loss rate, and the comparison of characteristic parameters *T*_p_, *S* and *R*_w_ are profiled in Fig. 9–11, respectively. From Fig. 9, the sharp decrease of DTG curves of the PU/ZnO/SOF mixture appear in the temperature range of 200–400 °C. Apparently, this mass loss is caused by the evaporation of the SOF, which is in the form of diesel and lubricating oil in this study. Meanwhile, the two oils have a few advantages in the combustion of the PU/ZnO mixture. However, the max mass loss rate *W*_max_ of PU/ZnO/SOF mixtures all decrease compared to the PU/ZnO mixture, and the starting temperature *T*_s_ of the two PU/ZnO/SOF mixtures declined in different ways. Significantly, the reduction of *T*_s_ of PU/ZnO/15W is 23 °C compared to PU/ZnO mixture, which is the largest reduction among the PU/ZnO/SOF mixtures. Fig. 10 compares the *T*_p_ among different PU/ZnO/SOF mixtures. After adding various SOF contents, the lowest peak temperature is still generated by PU/ZnO/15W at around 600 °C. The distinction may be illuminated by the fact that diesel fuel is a mixture of multiple hydrocarbons^48^ produced by crude oil while the lubricating oil is composed of base oil and several additives.^49^ The metal elements in the additives participate in the combustion to produce intermediates, which promote the combustion of the PU/ZnO mixture. From Fig. 11, the *S* and *R*_w_ indices of PU/ZnO/SOF mixtures all decrease compared to the PU/ZnO mixture. The change in the internal microstructure of soot particles may be responsible for this phenomenon.^50^ Except for evaporated oil, another part of oil was absorbed by the soot particles before the soot starts to burn. Due to the collision and coagulation of the ash content of the lubricating oil, the microcrystalline length of the soot particles increased and the degree of the carbon layer structure improved; thus, the particles do not easily decompose.^33^ However, the gap of combustion indices among soot/ash/SOF mixtures is not obvious. The largest value of *S* index is 1.024 × 10^−7^%^2^ min^−2^ °C^−3^ from the PU/ZnO/15W mixture, while the minimum value of *S* index is 1.009 × 10^−7^%^2^ min^−2^ °C^−3^ from the PU/ZnO/0# mixture. The difference of *S* is only 0.015 × 10^−7^%^2^ min^−2^ °C^−3^, which indicates that the difference of comprehensive combustion characteristics of soot/ash/SOF mixtures is not significant. Due to the small difference in comprehensive combustion characteristics, it is necessary to include the characteristic temperature to examine the combustion performance of the PU/ash/SOF mixtures. When the starting and peak temperatures are lower, the PU/ash/SOF mixture is more likely to ignite. For this reason, the 15W lubricating oil was chosen as the simulating SOF to perform the following experiments.

### 3.4 Influence of SOF proportion on soot combustion

**Fig. 12:**
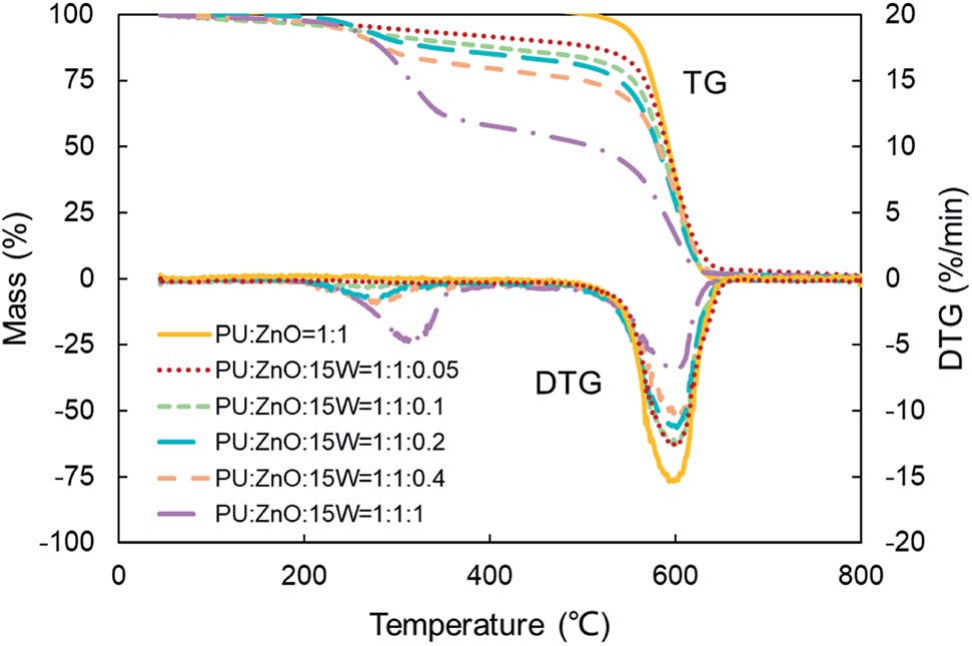
TG and DTG curves of combustion for soot/ash/SOF mixtures at various blending ratios.

**Fig. 13:**
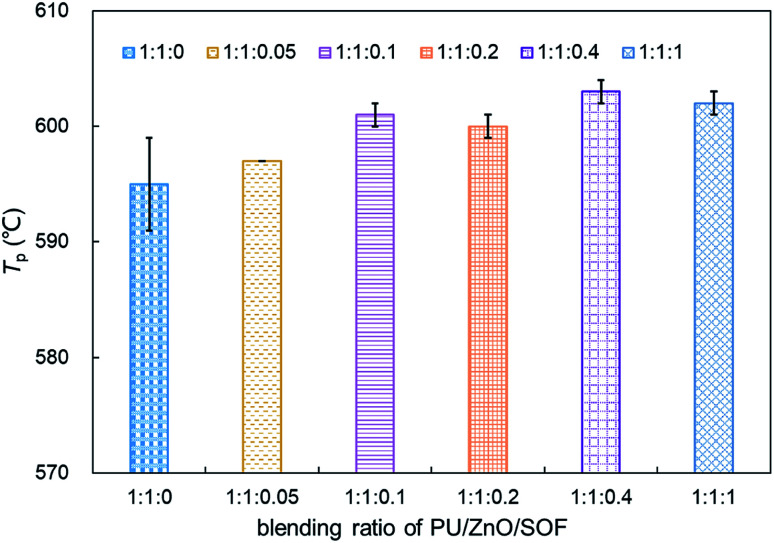
Comparison of the peak temperature *T*_p_ of soot/ash/SOF mixture at various blending ratios.

**Fig. 14:**
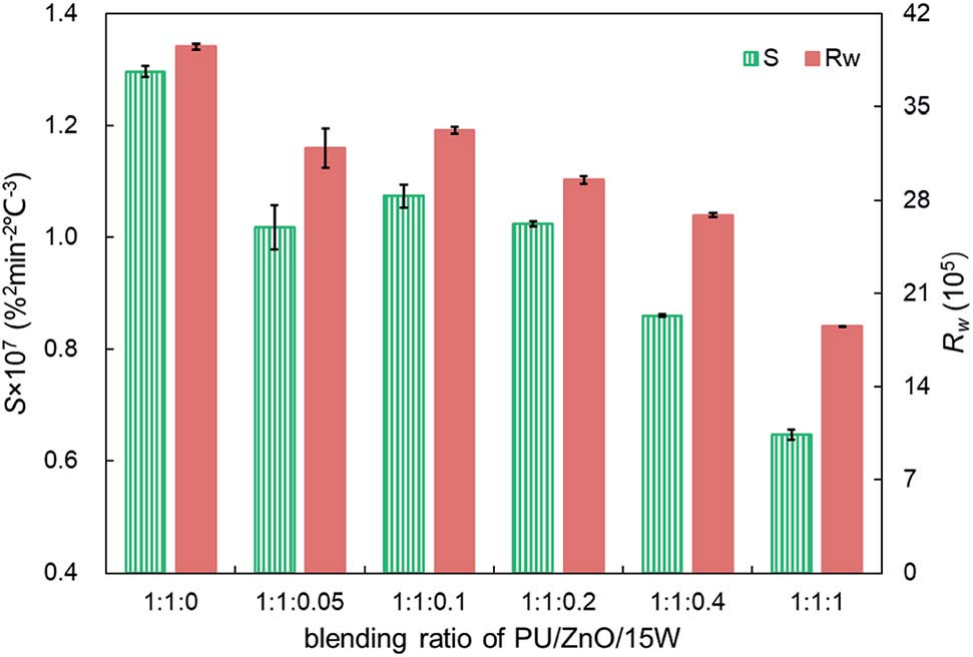
Comparison of the *S* and *R*_w_ indices for soot/ash/SOF mixtures at different blending ratios.

As shown in Fig. 12, the strong evaporation is connected to the increasing blending ratios of 15W lubricant, and the largest evaporation is obtained at the 1 : 1 : 1 weight ratio of the PU : ZnO : 15W mixture. When the proportion of SOF increases, the max mass loss rate *W*_max_ increases first then decreases continuously based on the DTG curves. When the weight ratio of the PU : ZnO : 15W mixture is 1 : 1 : 0.1, the *W*_max_ reaches the maximum value of 12.74% min^−1^, which is still lower than the PU/ZnO mixture. Instead, the *T*_e_ and *T*_s_ have little regular change. A comparison of the peak temperature *T*_p_ of soot/ash/SOF mixtures at different blending ratios is shown in Fig. 13. The *T*_p_ of PU/ZnO/15W fluctuates above and below 600 °C but all the values are higher than that of the PU/ZnO mixture, which indicates that the 15W lubricant has a negative effect on soot oxidation. This may be explained by the oxygen content of the lubricant which is higher, so the C and H have more opportunity to participate in the combustion process and in the oxygen reaction. This phenomenon prompts soot particles to crack into smaller sized particles, which leads to decreased levels in soot particle spacing and soot particles that are not easily oxidized.^33^ In addition, Fig. 14 compares the *S* and *R*_w_ indices for soot/ash/SOF mixtures at different blending ratios. From Fig. 14, the ensemble pattern is that the *S* and *R*_w_ indices are enhanced initially then decrease gradually with increasing ratios of SOF. When the weight ratio of the PU : ZnO : 15W mixture is 1 : 1 : 0.1, the largest values of *S* and *R*_w_ are 1.074 × 10^−7^%^2^ min^−2^ °C^−3^ and 33.29 × 10^5^, respectively, which is consistent with the variation of maximum loss rate *W*_max_. The possible reason is that the lubricating oil produces ash after combustion, and that over-loaded ash blocks the surface of soot particles and the active sites for O_2_ tend to be saturated so that soot combustion is reduced.^31^ Consequently, the 1 : 1 : 0.1 weight ratio of the soot/ash/SOF mixture is the optimal option after adding the SOF. In fact, the soot combustion will be better if no SOF participates in the reaction. However, it is difficult to always avoid the formation of SOF in the regeneration process, so more efforts are needed to control the use of lubricants.

## 4. Conclusions

The combustion characteristics of soot in the presence of ash and SOF are investigated by TGA in this study. The following major conclusions can be drawn based on this preliminary work.

(1) Compared to PU/SiO_2_, PU/Fe_2_O_3_, and PU/CaSO_4_ mixtures, the PU/ZnO mixture has the greatest effect on improving soot combustion because ZnO nanoparticles have an outstanding ability to carry oxygen.

(2) When the weight ratio of PU/ZnO is 1 : 1, ZnO has the most obvious promotion effect on soot combustion. Compared to pure PU, the comprehensive combustion index *S* and combustion stability index *R*_w_ increase from 0.667 × 10^−7^%^2^ min^−2^ °C^−3^ and 23.53 × 10^5^ to 1.296 × 10^−7^%^2^ min^−2^ °C^−3^ and 39.53 × 10^5^, respectively.

(3) With the addition of SOF, the comprehensive combustion index *S* and the combustion stability index *R*_w_ decrease due to changes in the microphysical properties of soot. As a simulated SOF, the 15W lubricant is superior to 0# diesel fuel in soot combustion to some degree.

(4) The interaction effect of SOF and ash is exhibited best at the 1 : 1 : 0.1 weight ratio of PU/ZnO/15W lubricant, in which the comprehensive combustion index *S* and combustion stability index *R*_w_ reach the maximum values of 1.074 × 10^−7^ and 33.29 × 10^5^%^2^ min^−2^ °C^−3^, respectively.

The interaction effect of ash and SOF on soot combustion was studied by TGA with an analysis of the combustion characteristic parameters. The results indicate that ZnO effectively promotes soot combustion while SOF has a negative impact on the soot combustion performance. The main significance of this work is to broaden the present understanding of soot combustion in the presence of ash and SOF. Undoubtedly, there are still more unexplored aspects of soot combustion. In particular, the interaction effect of SOF and catalyst on soot oxidation is worthy of future investigation.

## Conflicts of interest

There are no conflicts to declare.

## Supplementary Material
